# The association of *ACE1*, *ACE2*, *TMPRSS2*, *IFITM3* and *VDR* polymorphisms with COVID-19 severity: A systematic review and meta-analysis

**DOI:** 10.17179/excli2022-4976

**Published:** 2022-06-20

**Authors:** Zorana Dobrijevic, Dragana Robajac, Nikola Gligorijevic, Miloš Šunderic, Ana Penezic, Goran Miljuš, Olgica Nedic

**Affiliations:** 1 University of Belgrade - Institute for the Application of Nuclear Energy (INEP), Belgrade, Serbia

**Keywords:** COVID-19, ACE1, ACE2, TMPRSS2, IFITM3, VDR

## Abstract

Genes involved in the regulation of viral recognition and its entry into a host cell have been identified as candidates for genetic association studies on COVID-19 severity. Published findings on the effects of polymorphisms within *ACE1*, *ACE2*, *TMPRSS2*, *IFITM3* and *VDR* genes remained inconclusive, so we conducted a systematic review and meta-analysis in order to elucidate their potential involvement in the genetic basis underlying the severity of COVID-19 and/or an outcome of SARS-CoV-2 infection. Identification of potentially eligible studies was based on PubMed, Scopus and Web of Science database search. Relevant studies (n=29) with a total number of 8247 SARS-CoV-2-positive participants were included in qualitative synthesis, while results of 21 studies involving 5939 were pooled in meta-analysis. Minor allele I of rs1799752 located within *ACE1* was identified as a protective variant against severe COVID-19, while its effect on mortality rate was opposite. Similarly, minor allele A of *ACE2* polymorphism, rs2285666, was found to associate with a decreased risk of severe COVID-19 (*P* = 0.003, OR = 0.512, 95 % CI = 0.331-0.793). Statistical significance was also seen for the association between COVID-19 severity and rs12329760 located within *TMPRSS2*. Our results did not support the supposed association of rs12252 in *IFITM3* and polymorphisms within *VDR* with disease severity. We conclude that genetic variants within *ACE1*, *ACE2* and *TMPRSS2* may be potential biomarkers of COVID-19 severity, which needs to be further confirmed in a larger set of studies.

## Introduction

Substantial research efforts have been focused on the discovery of factors predisposing SARS-CoV-2-positive patients to develop a severe disease. Foundations for such investigations were discoveries related to molecular basis of SARS-CoV-2 infection, the immune response in COVID-19, and the observed similarities with other, previously characterized, respiratory infections (Jackson et al., 2022[25]; Zhang et al., 2022[58]). Receptors involved in the recognition of viral particles and their entry into host cells, as well as the components of signaling pathways initiated by the interaction of SARS-CoV-2 with corresponding receptors, have been identified as candidates in the search for genetic origins underlying severe clinical forms of COVID-19 (Deng et al., 2021[14]; Elhabyan et al., 2020[16]). 

Viral S protein interacts with receptors located on plasma membranes of host cells. Angiotensin converting enzyme 2 (ACE2), a member of the renin-angiotensin system, is the most efficient binding partner (Jackson et al., 2022[25]). Variants located within this gene were previously analyzed for their association with clinical characteristics and the outcome of SARS-CoV infection (Chan et al., 2005[11]; Chiu et al., 2004[12]), as well as with cardiovascular disorders and diabetic complications (Burrell et al., 2013[9]; Rahimi et al., 2014[42]), common comorbidities associated with adverse effects on COVID-19 clinical progression. The most commonly analyzed *ACE2* polymorphism in the context of COVID-19 severity is rs2285666, which is proposed to affect splicing of the primary transcript (Karakaş Çelik et al., 2021[26]; Gómez et al., 2020[20]; Möhlendick et al., 2021[34]; Novelli et al., 2020[36]; Pouladi et al., 2021[40]). 

Another member of renin-angiotensin system involved in SARS-CoV-2 infection is Angiotensin converting enzyme 1 (ACE1). The hypothetical mechanism relies on the activity of this enzyme in the conversion of angiotensin I to angiotensin II, which subsequently affects the expression and function of *ACE2*. Furthermore, a disbalance in ACE1 and ACE2 activities results in vasoconstriction, proliferation, inflammation, tissue injury with fibrosis, thrombotic events and edema, all related to COVID-19 complications (Gemmati et al., 2020[17]; Guo et al., 2020[22]). A frequently analyzed genetic variant located within *ACE1* is an insertion/deletion intronic polymorphism hypothesized to affect the level of ACE1 expression (Gemmati et al., 2020[17]).

The efficiency of viral entry into a host cell is highly dependent on the action of Transmembrane serine protease 2 (TMPRSS2), which cleaves S protein, allowing its fusion with the membrane. In addition, the activity of TMPRSS2 affects the function of Interferon-induced transmembrane protein 3 (IFITM3), which was identified as a potentially relevant factor in the control of SARS-CoV-2 infection, due to its known involvement in other viral diseases, as well as to its interaction with S protein (Prelli Bozzo et al., 2021[41]; Shi et al., 2021[51]). Thus, genetic variants influencing the function of TMPRSS2 and IFITM3 may be expected to contribute to the expression of severe symptoms of COVID-19, or may convey the protective effect.

Vitamin D, a commonly recommended supplement in the treatment of COVID-19, is among the most extensively studied regulators of the activity of renin-angiotensin system. Other mechanisms of its protective role in COVID-19 have been proposed, including its effects on the innate and acquired immune response (Getachew et al., 2021[18]). Since the function of vitamin D is dependent on its interaction with a receptor (VDR), genetic variants located within VDR gene were analyzed as contributory factors in genetic susceptibility to various human pathologies, including infectious diseases (Basit et al., 2013[8]; Teymoori-Rad et al., 2019[52]).

The main objective of the present study is to elucidate effects of naturally occurring variants within *ACE1*, *ACE2*, *TMPRSS2*, *IFITM3* and *VDR* genes on clinical severity of COVID-19 and/or the outcome of SARS-CoV-2 infection. Since two previous meta-analyses (Oscanoa et al., 2021[37]; Saengsiwaritt et al., 2022[46]) investigated the effect of *ACE1* indel polymorphism, we conducted an updated pooled analysis aiming to provide a more accurate assessment. Justification for the present study may be seen in the inclusion of almost twice as many articles on the effects of *ACE1* polymorphisms, compared to the recent meta-analysis (Saengsiwaritt et al., 2022[46]). In addition, we intended to provide the updated results on the effects of *TMPRSS2* and *ACE2* polymorphisms. To our knowledge, the present meta-analysis is the first to investigate the association of *VDR* and *IFITM3* polymorphisms with COVID-19 severity. 

## Material and Methods

The present systematic review and meta-analysis were carried out in accordance with the Preferred Reporting Items for Systematic Review and Meta-Analysis Protocols (PRISMA) (Shamseer et al., 2015[50]).

### Publication search

The search strategy for potentially relevant studies in electronic literature databases PubMed, Web of Science and Scopus included combinations of keywords: gene/protein name or identifier and aliases (“Vitamin D receptor” or “VDR”; “Angiotensin converting enzyme” or “ACE” or “ACE1” or “CD143” or “DCP1”; “ACE2” or “ACEH”; “Transmembrane serine protease 2” or “TMPRSS2” or “PRSS10”; “Interferon induced transmembrane protein 3” or “IFITM3” or “DSPA2b”); term “polymorphism” or “genetic variant” and term “COVID-19” or “SARS-CoV-2”. Language restriction was not applied during the search. In order not to miss potentially suitable articles, we thoroughly examined reference lists of the retrieved original articles, as well as previously published reviews and meta-analyses. The selection was limited to articles published in 2020 and 2021 (publication date: database inception to December 31^st^ 2021).

### Inclusion/exclusion criteria, data extraction

Eligibility assessment of the retrieved studies was based on the following criteria: a) a study assessed differences in genotype distributions/allele frequencies of polymorphisms located within relevant genes (*VDR*, *ACE1*, *ACE2*, *TMPRSS2* or *IFITM3*) between groups of SARS-CoV-2-positive participants with different COVID-19 severity and/or outcome; b) original full-text articles (including short communications or brief reports, if methodology and results were presented in detail); c) sufficient data on genotype/allele counts for the calculation of risk estimates; d) detailed information provided about the recruitment of patients, diagnostic procedure, severity assessment criteria, outcome assessment, ethnicity of participants, genotyping and statistical methodology and other relevant data; e) minimum 50 SARS-CoV-2-positive participants. Studies which provided genotyping data for children and adults separately were included in qualitative and quantitative data synthesis taking into account only results for adults. 

Ecological studies were not considered relevant for this meta-analysis. Exclusion criteria were: retraction of articles and poor quality of the study design (obvious major errors introducing bias). Separate entries for each study were made for quantitative data synthesis in order to assess all possible comparisons between severity groups, together with fatality rates or other potential outcomes. 

The data relevant for the extraction from the selected studies included: first author's name and the year of article publication, country and ethnicity of participants, study design, criteria for the diagnosis of COVID-19, severity assessment criteria, patient recruitment method, identifiers of the analyzed genetic variants, genotyping methodology, sample size, age and gender of participants, genotype/allele counts. We reclassified patient groups based on COVID-19 severity in order to match the World Health Organization guidelines, since severity categories differed substantially between studies. Data synthesis was conducted if the results from three or more studies corresponded to a specific comparison of genotype distributions, aiming to evaluate association of a single genetic variant with disease severity/outcome. 

Methodological quality of studies included in qualitative and quantitative data synthesis was evaluated using Newcastle-Ottawa Scale, which considers three domains (selection of subjects, comparability and outcome) with the highest possible score of 9 (Wells et al., 2021[56]). Scoring for each study was based on the methodology related to SARS-CoV-2-positive participants, since this data synthesis is related to disease severity, even though the study design might have included healthy controls. 

### Statistical analysis

Statistical software OpenMeta-analyst (The Center for Evidence-based Medicine, Brown University, Providence, RI, USA) and MetaGenyo (Pfizer-University of Granada-Junta de Andalucía Centre for Genomics and Oncological Research, (GENYO), Granada, Spain) were used for assessing between-study heterogeneity and for conducting quantitative data synthesis (Martorell-Marugan et al., 2017[32]; Wallace et al., 2012[54]). For each entry in data-synthesis, as well as for the pooled effects, odds ratios (ORs) and their 95 % confidence intervals (CIs) were used as risk estimates. Between-study heterogeneity was determined using Cochran's Q test and the inconsistency index (I^2^), with *P* value <0.1 or I^2^≥50 % indicating significant heterogeneity. In cases when the results of heterogeneity tests were insignificant, Mantel-Haenszel method was used for pooling risk estimates under the fixed-effect model (Mantel and Haenszel, 1959[30]). Otherwise, random-effects statistical model was selected and a method proposed by DerSimonian and Laird (1986[15]) was used for pooling. Multiple testing was conducted for different genetic models of association. 

In cases when the number of entries for a single comparison was more than 5, potential publication bias was evaluated by the visual inspection of funnel plots and by using Egger's test. Robustness of the results of data synthesis was evaluated by sensitivity analysis (leave-one-out method). 

## Results

### Study identification 

**Figure 1:**
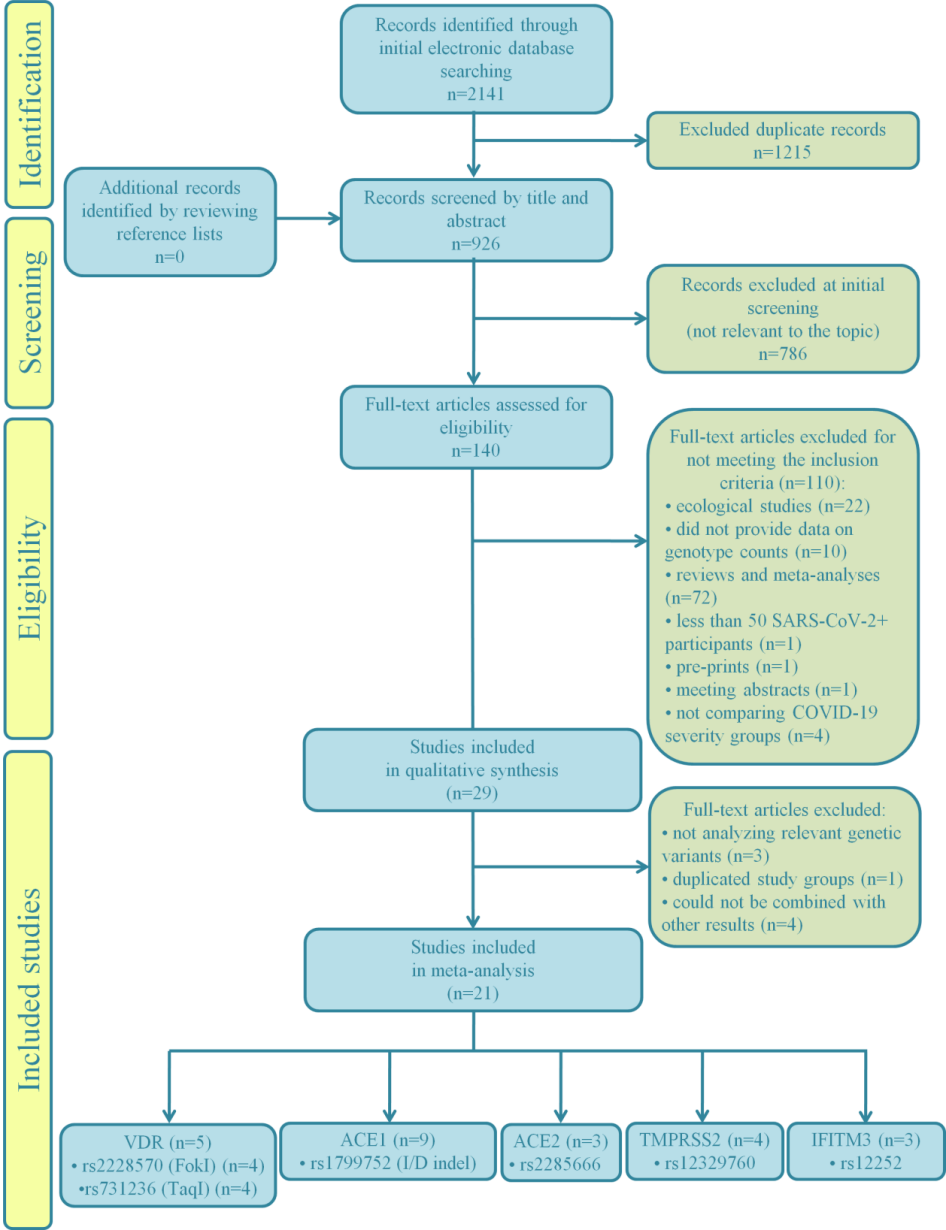
Flowchart of the study selection process

The study selection process for the present meta-analysis is illustrated by the flow chart given in Figure 1(Fig. 1). A total of 2141 records were retrieved in the initial phase of database search, 1215 of which were marked as duplicates and excluded from further screening procedure. Additionally, 786 records not relevant to the topic were removed after screening by the title and abstract. Among the remaining records, 94 were excluded due to inadequate study design (72 meta-analyses and reviews, 22 ecological studies). One article was a pre-print, not submitted to peer-review, while another record was a meeting abstract. We also excluded 3 studies which did not compare genotype distributions between patients stratified into severity groups, but compared patients with severe COVID-19 and healthy individuals instead. One study was excluded as it provided results only for patients with severe disease, while one more was excluded for not reaching a predefined minimum number of participants. Furthermore, 10 records were eliminated from subsequent data synthesis since they did not provide genotype/allele counts. 

Basic characteristics of the remaining 29 studies (Abdollahzadeh et al., 2021[1]; Aladag et al., 2021[4]; Al-Anouti et al., 2021[5]; Alghamdi et al., 2021[6]; Akbari et al., 2022[2]; Akin et al., 2022[3]; Apaydin et al., 2022[7]; Cafiero et al., 2021[10]; Karakaş Çelik et al., 2021[26]; Cuesta-Llavona et al., 2021[13]; Gómez et al., 2020[20]; Gómez et al., 2021[19]; Gunal et al., 2021[21]; Hubacek et al., 2021[23]; Íñiguez et al., 2021[24]; Kotur et al., 2021[27]; Kouhpayeh et al., 2021[28]; Mir et al., 2021[33]; Möhlendick et al., 2021[34]; Monticelli et al., 2021[35]; Novelli et al., 2020[36]; Peralta et al., 2021[39]; Ravikanth et al., 2021[43]; Saad et al., 2021[45]; Schönfelder et al., 2021[49][48]; Verma et al., 2021[53]; Wang et al., 2022[55]; Wulandari et al., 2021[57]; Zhang et al., 2020[59]), with a total number of 8247 SARS-CoV-2-positive participants, included in qualitative data synthesis, are given in Table 1(Tab. 1) (References in Table 1: Abdollahzadeh et al., 2021[1]; Akbari et al., 2022[2]; Akin et al., 2022[3]; Aladag et al., 2021[4]; Al-Anouti et al., 2021[5]; Alghamdi et al., 2021[6]; Apaydin et al., 2022[7]; Cafiero et al., 2021[10]; Cuesta-Llavona et al., 2021[13]; Gómez et al. , 2020[20], 2021[19]; Gunal et al., 2021[21]; Hubacek et al., 2021[23]; Íñiguez et al., 2021[24]; Karakaş Çelik et al., 2021[26]; Kotur et al., 2021[27]; Kouhpayeh et al., 2021;[28] Mir et al., 2021[33]; Möhlendick et al., 2021[34]; Monticelli et al., 2021[35]; Peralta et al., 2021[39]; Ravikanth et al., 2021[43]; Saad et al., 2021[45]; Schönfelder et al., 2021[49][48]; Verma et al., 2021[53]; Wang et al., 2022[55]; Wulandari et al., 2021[57]; Zhang et al., 2020[59]). Although 13 studies had case-control design, the presented data referred to COVID-19-positive participants, since the results regarding disease severity were relevant for this data synthesis. The majority of eligible studies included Caucasian patients (n=21). Results of the study quality assessment, based on Newcastle-Ottawa scale, indicated that studies were of high quality (score 7-9) except for two cases (score 6) of moderate quality (Supplementary Table 1). 

Among the selected studies, the majority analyzed polymorphisms within *ACE1* (n=13). However, not all of them were included in quantitative data synthesis, since one study (Íñiguez et al., 2021[24]) investigated variants other than the most common rs1799752. Furthermore, data on *ACE1* polymorphism reported in three studies could not be combined with the results from other articles, due to differences in stratification criteria applied to define patients' groups (Akbari et al., 2022[2]; Cafiero et al., 2021[10]; Hubacek et al., 2021[23]). 

Since rs2285666 was the only genetic variant in *ACE2* investigated in more than three studies reporting data which enabled severity/outcome comparison, two studies (Akin et al., 2022[3]; Wang et al., 2022[55]) which omitted to analyze this variant were excluded from meta-analysis. Other two studies were eliminated from data-synthesis related to *ACE2* for the same reason, but were kept in the selection of studies since they provided data on polymorphisms in other relevant genes. Thus, the final number of studies on ACE2 polymorphism rs2285666 included in meta-analysis was 3. 

As for *TMPRSS2*, there were 4 eligible studies with enough data to conduct data synthesis of results regarding polymorphism rs12329760. Although Monticelli et al. (2021[35]) did not provide complete data on genotype counts, their results were included in meta-analysis of the association of rs12329760 with COVID-19 severity under dominant genetic model. 

Results on the association of rs12252 located within IFITM3 with disease severity were extracted from 4 studies. A study of Gómez et al. (2021[19]) was excluded from quantitative data synthesis, since the same group of participants was included in the later study conducted by Cuesta-Llavona et al. (2021[13]). Furthermore, findings of Alghamdi et al. (2021[6]) could not be combined with the results of other studies since the segregation of patients into groups based on COVID-19 severity significantly differed.

Genetic variants within VDR were analyzed in 5 eligible studies, which provided enough data for the inclusion in meta-analysis for two polymorphisms, rs2228570 (FokI) and rs731236 (TaqI). 

### Quantitative data synthesis

The overall number of SARS-CoV-2-positive patients from studies included in quantitative synthesis was 5939. Various articles provided data on multiple genetic variants, as well as for multiple groups of patients stratified according to the outcome of SARS-CoV-2 infection, COVID-19 severity and/or lethal outcome, which were used as separate entries in meta-analysis (Table 1(Tab. 1)). The number of participants in the studies included in our meta-analysis for rs1799752 in *ACE1* was 1734, 656 for rs2285666 in *ACE2*, 2021 for rs12329760 in *TMPRSS2*, 803 for rs12252 in *IFITM3*, 1516 for rs2228570 and 1547 for rs731236 in *VDR*. 

**Figure 2:**
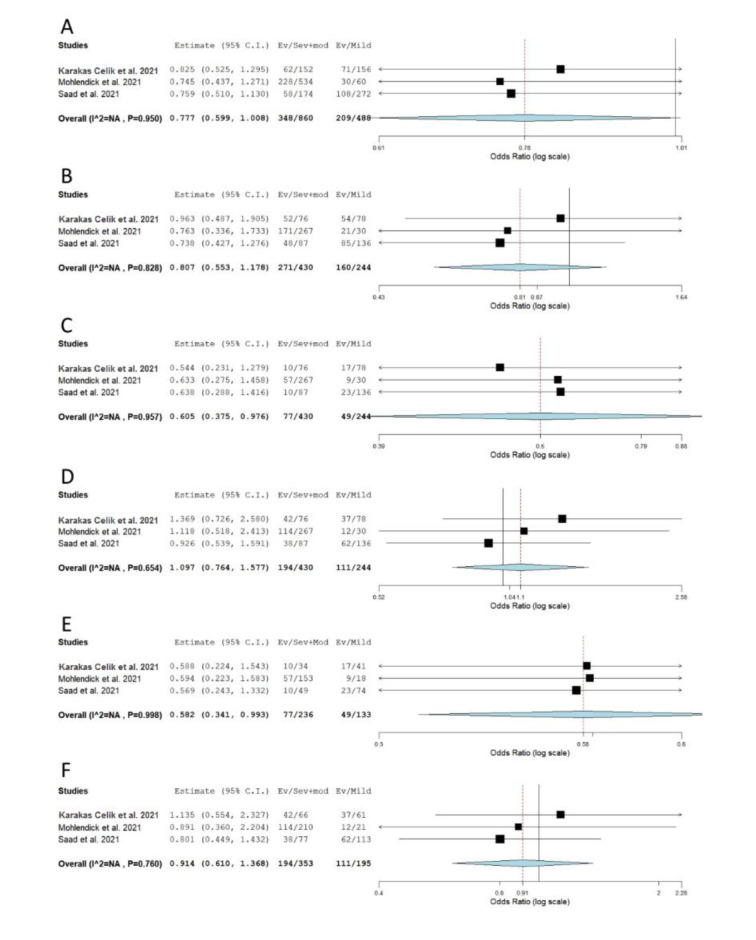
Meta-analysis of the association between rs1799752 in *ACE1* and COVID-19 severity: comparison severe+moderate vs. mild. A) allelic model; B) dominant model; C) recessive model; D) overdominant model; E) II vs. DD; F) DI vs. DD. The results of the included studies presented as ORs, with 95 % CI, and the overall effect with 95 % CI are shown in the forest plot. *P* values given are derived from heterogeneity tests.

**Figure 3:**
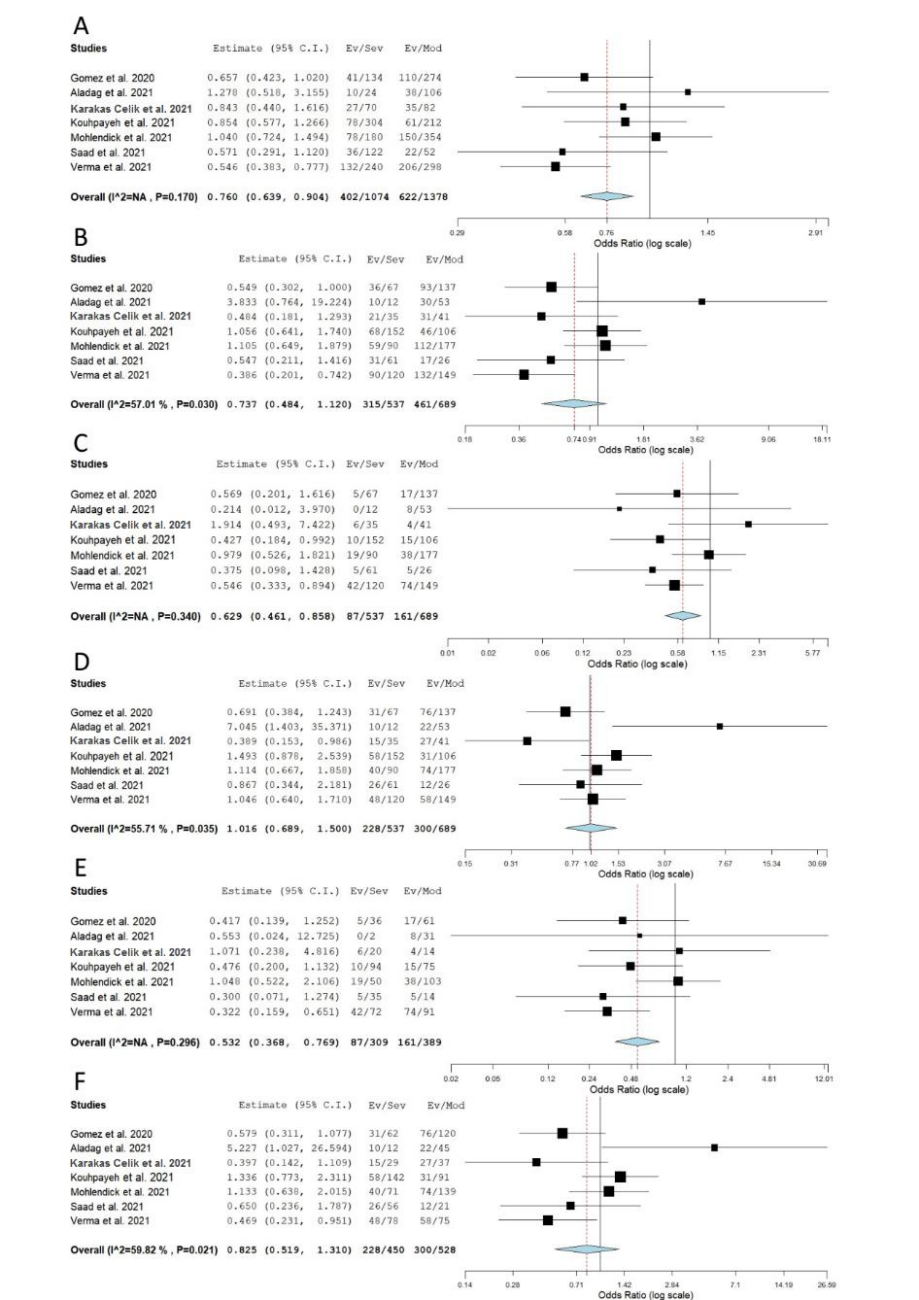
Meta-analysis of the association between rs1799752 in *ACE1* and COVID-19 severity: comparison severe vs. moderate. A) allelic model; B) dominant model; C) recessive model; D) overdominant model; E) II vs. DD; F) DI vs. DD. The results of the included studies presented as ORs, with 95 % CI, and the overall effect with 95 % CI are shown in the forest plot. *P* values given are derived from heterogeneity tests.

**Figure 4:**
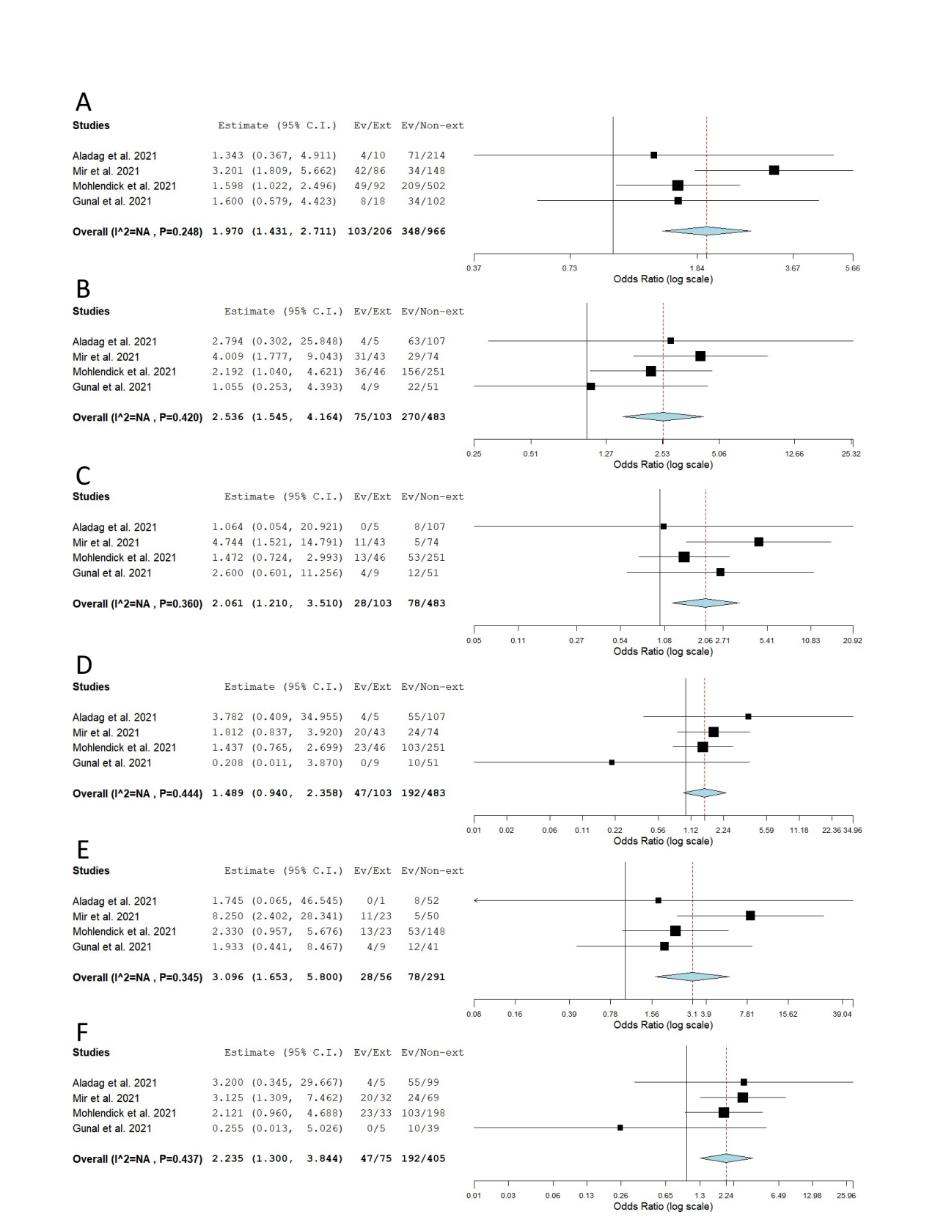
Meta-analysis of the association between rs1799752 in *ACE1* and COVID-19 outcome: comparison exitus vs. survivors. A) allelic model; B) dominant model; C) recessive model; D) overdominant model; E) II vs. DD; F) DI vs. DD. The results of the included studies presented as ORs, with 95 % CI, and the overall effect with 95 % CI are shown in the forest plot. *P* values given are derived from heterogeneity tests.

**Figure 5:**
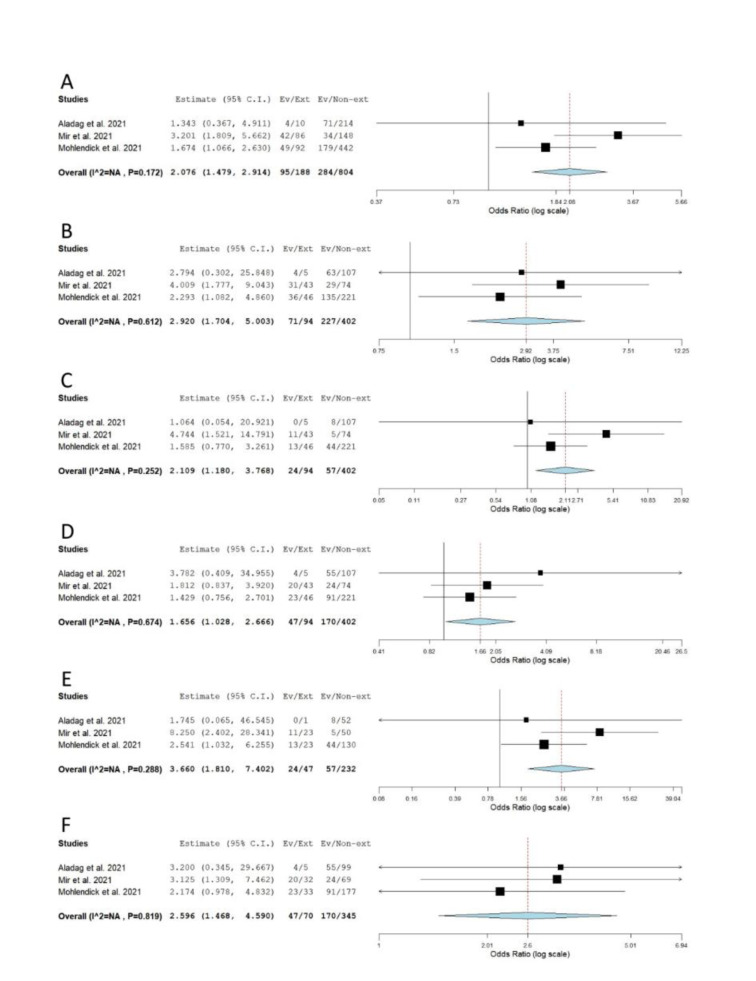
Meta-analysis of the association between rs1799752 in *ACE1* and COVID-19 outcome in hospitalized patients: comparison exitus vs. survivors. A) allelic model; B) dominant model; C) recessive model; D) overdominant model; E) II vs. DD; F) DI vs. DD. The results of the included studies presented as ORs, with 95 % CI, and the overall effect with 95 % CI are shown in the forest plot. *P* values given are derived from heterogeneity tests.

When genotype distributions of rs1799752 were compared between COVID-19 patients with severe and non-severe disease, the results remained statistically insignificant for all genetic models tested (Table 2(Tab. 2); Reference in Table 2: Monticelli et al., 2021[35]; Supplementary Figure 1). However, minor allele I homozygotes were found associated with lower disease severity, when genotype frequencies of rs1799752 were compared between patients with severe/moderate and mild form of COVID-19 (*P*_rec_ = 0.039, OR_rec_= 0.605, 95 % CI = 0.375-0.976; *P*_II vs. DD _= 0.047, OR_II vs. DD _= 0.582, 95 % CI = 0.341-0.993) (Table 2(Tab. 2), Figure 2(Fig. 2); References in Figure 2: Karakaş Çelik et al., 2021[26]; Möhlendick et al., 2021[34]; Saad et al., 2021[45]). Furthermore, among patients hospitalized with severe or moderate illness, minor allele I was found to associate with lower COVID-19 severity (Table 2(Tab. 2), Figure 3(Fig. 3); References in Figure 3: Aladag et al., 2021[4]; Gómez et al. , 2020[20]; Karakaş Çelik et al., 2021[26]; Kouhpayeh et al., 2021[28]; Möhlendick et al., 2021[34]; Saad et al., 2021[45]; Verma et al., 2021[53]). Contrary to these results, minor allele I was shown to confer an increased risk of lethal outcome in COVID-19 patients. Statistically significant results were found for multiple genetic models of association (*P*_allelic_< 0.001; *P*_dom_< 0.001; *P*_rec _= 0.008, OR_rec _= 2.061; *P*_II vs. DD _< 0.001; *P*_DI vs. DD _= 0.004) (Table 2(Tab. 2), Figure 4(Fig. 4); References in Figure 4: Aladag et al., 2021[4]; Gunal et al., 2021[21]; Mir et al., 2021[33]; Möhlendick et al., 2021[34]). Similar results were obtained when COVID-19 fatality rate was assessed in hospitalized patients (Table 2(Tab. 2), Figure 5(Fig. 5); References in Figure 5: Aladag et al., 2021[4]; Mir et al., 2021[33]; Möhlendick et al., 2021[34]).

**Figure 6:**
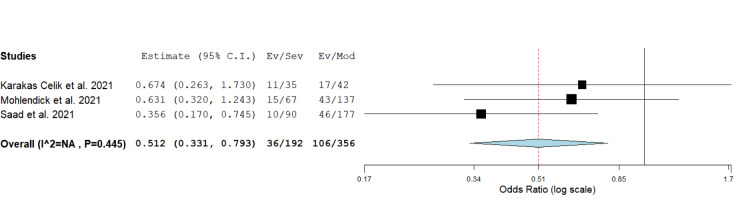
Meta-analysis of the association between rs2285666 in *ACE2* and COVID-19 severity under the dominant genetic model (carriers vs. non-carriers): comparison severe vs. moderate. The results of the included studies presented as ORs, with 95 % CI, and the overall effect with 95 % CI are shown in the forest plot. *P* values given are derived from heterogeneity tests.

Since genotype counts for rs2285666 were available only for minor allele A carriers (regardless of their gender) in the required number of studies, we performed the analysis for the dominant genetic model (GA+AA vs. GG in females, A vs. G in males). The results suggested that allele A is protective against development of a severe form of COVID-19 (severe vs. moderate disease comparison, *P* = 0.003, OR = 0.512, 95 % CI = 0.331-0.793) (Table 2(Tab. 2), Figure 6(Fig. 6); References in Figure 6: Karakaş Çelik et al., 2021[26]; Möhlendick et al., 2021[34]; Saad et al., 2021[45]). There were insufficient data for separated analysis of the effect of rs2285666 in female and male gender, as well as for testing different genetic models in female patients.

**Figure 7:**
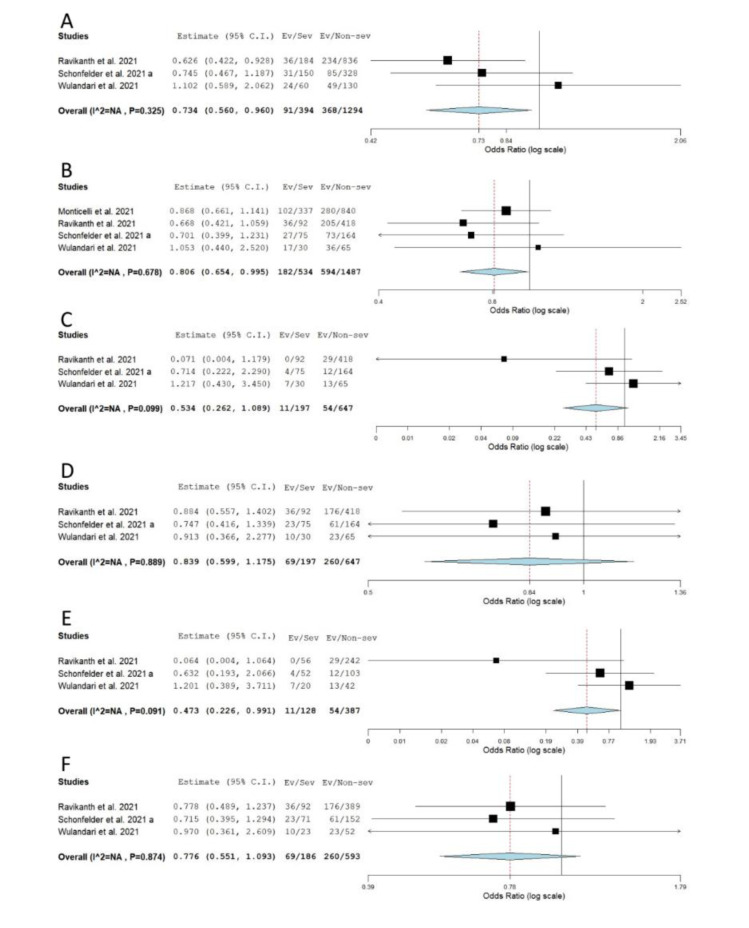
Meta-analysis of the association between rs12329760 in *TMPRSS2* and COVID-19 severity: comparison severe vs. non-severe. A) allelic model; B) dominant model; C) recessive model; D) overdominant model; E) AA vs. GG; F) GA vs. GG. The results of the included studies presented as ORs, with 95 % CI, and the overall effect with 95 % CI are shown in the forest plot. *P* values given are derived from heterogeneity tests.

When the association between rs12329760 and COVID-19 severity was tested, statistical significance was reached for allelic, recessive and dominant genetic models (severe vs. non-severe comparison). The obtained results suggested that minor allele A confers a protective effect against the progression of COVID-19 to a severe form (*P*_allelic_ = 0.024, OR_allelic _= 0.734, 95 % CI = 0.560-0.960; *P*_dom_ = 0.045, OR_dom _= 0.806, 95 % CI = 0.654-0.995; *P*_rec_ = 0.047, OR_rec _= 0.473, 95 % CI = 0.226-0.991) (Table 2(Tab. 2), Figure 7(Fig. 7); References in Figure 7: Monticelli et al., 2021[35]; Ravikanth et al., 2021[43]; Schönfelder et al., 2021[49]; Wulandari et al., 2021[57]). However, statistical significance was lost after the exclusion of asymptomatic cases from comparisons (*P*_allelic_ = 0.553; *P*_rec_ = 0.560) (Table 2(Tab. 2), Supplementary Figure 2).

When genotype distributions between patients with severe and moderate illness were compared, no association of rs12252 with COVID-19 severity was seen in any comparison (Table 2(Tab. 2), Supplementary Figure 3). Similarly, no significant relations were determined when genotype frequencies of rs2228570 and rs731236 were compared between patients with severe and non-severe illness (Table 2(Tab. 2), Supplementary Figures 4 and 5). When ethnic subgrouping of participants was performed, only the analysis of the effect of rs2228570 in Caucasians could be conducted (3 studies), but the results remained statistically insignificant (Table 2(Tab. 2), Supplementary Figure 4). 

### Publication bias assessment and sensitivity analysis

Publication bias was assessed only for studies on rs1799752 in severely and moderately ill patients. The visual inspection of Funnel plots, together with the corresponding *P* values, did not suggest the presence of publication bias (Supplementary Figure 6). In sensitivity analysis for the same group of studies, leaving out the study conducted by Verma et al. (2021[53]) resulted in the loss of statistical significance for allelic and recessive genetic models (Supplementary Figure 7). The results of the present meta-analysis were, however, shown to be statistically stable for II vs. DD comparison, since the exclusion of studies did not significantly affect the pooled ORs (Supplementary Figure 7). 

## Discussion

The majority of studies included in the present data synthesis analyzed genetic variant rs1799752 located within *ACE1*. Even though this polymorphism is intronic, it was shown that the presence of Alu insertion (I allele) correlates with the lower expression of ACE1, possibly via regulation of the transcription rate (Mafra et al., 2018[29]; Mariner et al., 2008[31]; Rigat et al., 1990[44]). The protective effect of the same allelic variant against severe COVID-19 was shown in Spanish and Turkish COVID-19 patients (Aladag et al., 2021[4]; Gómez et al., 2020[20]). Furthermore, Kouhpayeh et al. (2021[28]) demonstrated that a reduced risk of developing severe COVID-19 was associated with I allele under the assumed recessive genetic model. The protective effect of this allele was also reported in COVID-19 patients from Lebanon and India (Saad et al., 2021[45]; Verma et al., 2021[53]). Contrary to these results, Karakaş Çelik et al. (2021[26]), Möhlendick et al. (2021[34]) and Akbari et al. (2022[2]) failed to confirm a supposed association between rs1799752 and disease severity. In their study conducted on Saudi Arabian patients, Mir et al. (2021[33]) claimed that minor allele I has protective effect against disease severity. Their results, however, could not be included in our data synthesis, since genotype distributions were compared between COVID-19 cases and healthy individuals (Mir et al., 2021[33]).

When the presentation of symptoms was analyzed as a criterion for the assessment of the association of rs1799752 with the severity of SARS-CoV-2 infection, Cafiero et al. (2021[10]) reported that a reduced risk of the development of symptomatic disease was associated with minor allele I. II genotype was also found to be predominantly present in asymptomatic participants in the study conducted by Gunal et al. (2021[21]). 

Our meta-analysis demonstrated the association between genetic variant rs1799752 located within *ACE1* and COVID-19 severity for multiple genetic models tested. According to the obtained results, the minor allele I confers a decreased risk of developing severe clinical symptoms. These findings are not surprising, since the majority of studies included in the pooled analysis demonstrated a protective effect of allele I or II genotype, although the authors applied different criteria for the assessment of disease severity. The association with severe COVID-19 was found when patients with severe and moderate illness were compared, most of them being hospitalized for COVID-19. Also, the protective effect of II genotype was confirmed when genotype distributions between patients with mild disease and those with clinically more relevant symptoms were compared. Although statistical significance was not found in the severe vs. non-severe COVID-19 comparison, the obtained results could be explained by differences in the patient recruitment and classification methods.

Our results are in accordance with those reported in previous meta-analysis by Saengsiwaritt et al. (2022[46]), where the minor allele I was used as referent in data presentation. However, authors made a major error in their data-synthesis by not recognizing rs1799752 and rs4646994 as synonyms for the same genetic variant and, therefore, excluding several eligible studies from the pooled analysis. Our results are also consistent with those obtained by Oscanoa et al. (2021[37]) in their meta-analysis which included only five studies on the effects of rs1799752 and different patient classification criteria. 

Gunal et al. (2021[21]) and Aladag et al. (2021[4]) did not find statistically significant association between rs1799752 and COVID-19 fatality in their groups of patients from Turkey. Still, it should be noted that the number of heterozygous genotypes in the study by Gunal et al. (2021[21]) was much lower than expected according to allelic frequencies. Since a genotyping method applied in their study was previously associated with genotyping errors in the case of rs1799752, results obtained using the same methodology need to be taken with caution. Other analyses, which used two rounds of PCR, were designed to avoid miscalling of heterozygous genotypes for DD homozygotes (Saracevic et al., 2013[47]). In their study on Saudi Arabian patients, Mir et al. (2021[33]) claimed that they had found a protective effect of the minor allele I against lethal outcome of COVID-19. Again, when we analyzed genotype frequencies, an error in the assessment of risk estimates was noticed, suggesting an opposite effect of the same allelic variant. 

The present meta-analysis showed the association of rs1799752 with a lethal outcome of COVID-19. Since studies included in the analysis varied in their design and in the inclusion of patients with mild disease, we conducted a second round of data synthesis with data only on hospitalized COVID-19 patients. The results of two analytical approaches were similar, but higher OR values were obtained when hospitalized participants were considered alone. However, the minor allele I was found to be associated with an increased susceptibility to lethal outcome, which is in contrast to the results found in a test of its association with disease severity. Explanations for such disagreement may be searched in a relatively low number of participants in the group of deceased patients with COVID-19, in presence of comorbidities that significantly influenced the results of association studies, as well as in differences in transcriptional and posttranscriptional regulation of the expression of ACE1 between a disease and healthy condition. In their study on Greek population, Papadopoulou et al. (2022[38]) found differences in ACE1 activity related to genotypes between patients with COVID-19 and control subjects, and suggested a more complex and disease-specific relation between ACE1 genotypes, activity of ACE1 and disease progression. Our results could not be compared with previously published ones, since this is the first pooled analysis on the effect of rs1799752 on COVID-19 lethality.

As for the genetic variant rs2285666 in *ACE2*, Gómez et al. (2020[20]) and Karakaş Çelik et al. (2021[26]) found no statistically significant difference in the frequency of the minor allele A between their patients with severe COVID-19 and other hospitalized patients. On the other hand, Möhlendick et al. (2021[34]) stated that minor allele A exhibited a protective effect against disease severity and mortality in the group of patients from Germany. However, their results contained major, yet common, error since hemizygous genotypes were misinterpreted as homozygotes and combined with genotyping results for female patients. Saengsiwaritt et al. (2022[46]) failed to identify that error, so they conducted meta-analyses for different genetic models, although the results allowed testing only for the dominant model. Our results suggesting a protective effect of rs2285666 minor allele A against disease severity in COVID-19 patients are, thus, not entirely comparable with the previously published.

In their analysis on the association between rs12329760 in *TMPRSS2* and the risk of severe COVID-19, Monticelli et al. (2021[35]) reported a protective effect of the minor allele A. Ravikanth et al. (2021[43]) announced similar findings, since they noticed a lower frequency of the minor allele in asymptomatic SARS-CoV-2-infected persons compared to the most severely affected COVID-19 patients. Contrary to these results, no association between rs12329760 and the disease severity was found in two other studies (Schönfelder et al., 2021[49]; Wulandari et al., 2021[57]). Taking into account the size of the population in each study, the pooled statistically significant results were most probably greatly influenced by two large studies (Monticelli et al., 2021[35]; Ravikanth et al., 2021[43]). Furthermore, differences in the results obtained after the exclusion of asymptomatic participants indicate that the acquired associations need to be taken with caution. 

No association between rs12252 in *IFITM3* and a risk of developing severe COVID-19 was found in a relatively large study on Spanish population (Cuesta-Llavona et al., 2021[13]). Similar results were obtained by Schönfelder et al. (2021[48]), although their group of non-severe patients included non-hospitalized cases with mild disease as well. The findings of Zhang et al. (2020[59]) were statistically insignificant unless adjusted for age. Therefore, the lack of statistical significance in data synthesis was expected, based on the results of individual eligible studies. 

In the case of polymorphisms within *VDR*, the meta-analysis was possible for the association of rs2228570 and rs731236 with COVID-19 severity. Apaydin et al. (2022[7]) stated that heterozygous genotype of rs2228570 was associated with an increased disease severity, while such association was not found for rs731236. Two VDR polymorphisms included in the present data synthesis were not among variants found to associate with severe COVID-19 in a large study conducted in the UAE (Al-Anouti et al., 2021[5]), or in studies conducted on Iranian, Serbian and Cuban COVID-19 patients (Abdollahzadeh et al., 2021[1]; Kotur et al., 2021[27]; Peralta et al., 2021[39]). Still, Abdollahzadeh et al. (2021[1]) observed the association of rs2228570 with symptomatic SARS-CoV-2 infection. The results of our meta-analysis regarding rs2228570 remained statistically insignificant for all applied genetic models of association, even after stratification according to ethnicity of the participants.

## Conclusions

The results of the present meta-analysis clearly qualify rs1799752 for a genetic variant associated with COVID-19 severity. Since the number of studies included in this meta-analysis of the effects of all polymorphisms tested is relatively low, a caution is suggested when considering the obtained results. Another limitation of our meta-analysis is a small number of patients in certain severity- or outcome-based groups. Therefore, additional studies with larger sample size are needed to confirm the significance of the associations found in the current data synthesis, including those on the effects of *ACE2* and *TMPRSS2* polymorphisms. Further studies are also needed in order to assess the potential influence of ethnicity, as well as to investigate gender differences related to the effect of variants, especially those located on X-chromosome. Additional data would also provide means for testing effects of the examined polymorphisms on specific parameters related to clinical progression of COVID-19, such as presentation of clinical symptoms or COVID-19-related mortality. Future studies should test associations of other genetic variants which were not included in this data synthesis. Finally, it would be highly relevant to test joint effects of all risk variants identified in the present meta-analysis, since genes of interest are functionally related.

## Declaration

### Acknowledgments

The research was supported by the Ministry of Education, Science and Technological Development of the Republic of Serbia (Agreement no. 451-03-68/2020-14/200019).

### Author contributions

ZD: Conceptualization, investigation, formal analysis, visualization, writing - original draft. DR: Investigation, formal analysis, visualization, writing - review & editing. NG: Investigation, formal analysis, visualization, writing - review & editing. MŠ: Investigation, formal analysis, writing - review & editing. AP: Investigation, formal analysis, writing - review & editing. GM: Investigation, formal analysis, writing - review & editing. ON: Validation, supervision, project administration, funding acquisition, writing - review & editing. 

### Conflict of interest 

The authors declare that they have no conflict of interest.

### Data availability 

The data supporting the findings of this study are available within the article, its supplementary materials, or from the corresponding author, upon reasonable request. 

## Supplementary Material

Supplementary information

## Figures and Tables

**Table 1:**
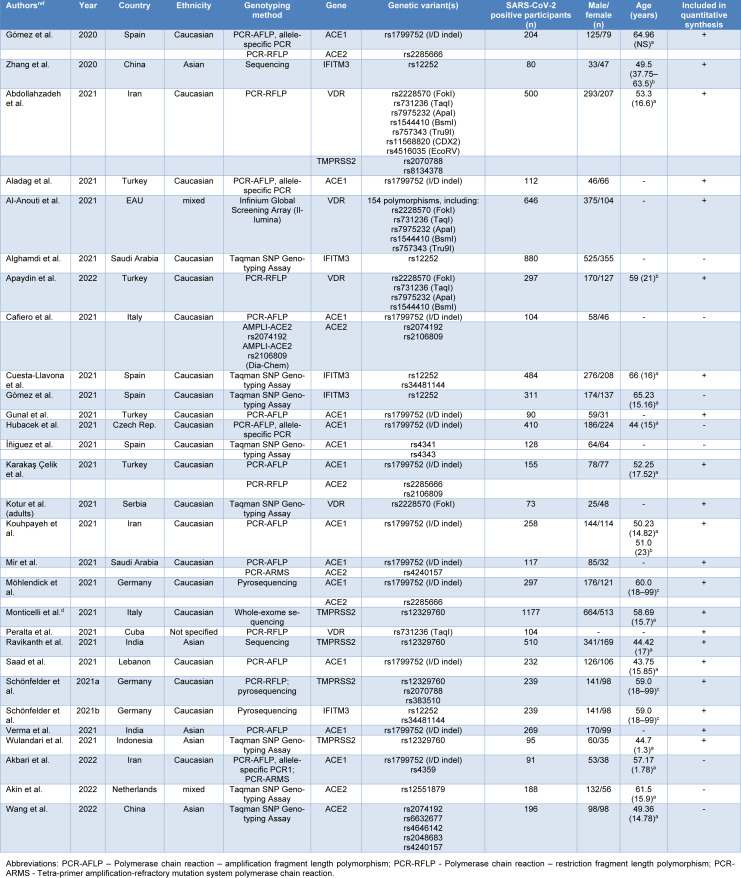
Characteristics of the studies included in the qualitative synthesis

**Table 2:**
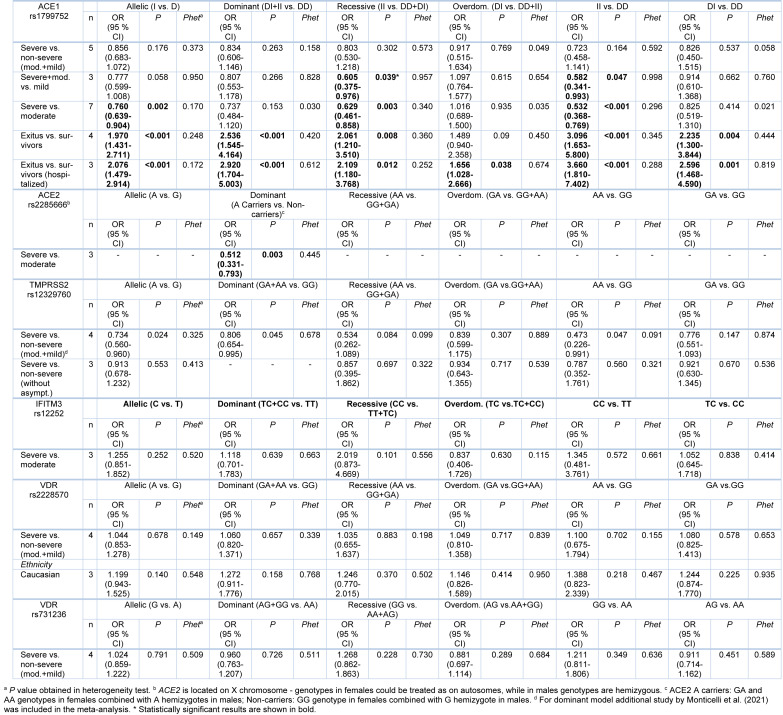
Meta-analysis of association between genetic variants in *ACE1*, *ACE2*, *TMPRSS2*, *IFITM3* and *VDR* and COVID-19 severity
