# Supplementary material for: The association of ACE1, ACE2, TMPRSS2, IFITM3 and VDR polymorphisms with COVID-19 severity: A systematic review and meta-analysis
Source: EXCLI J. 2022 Jun 20;21:818–39. doi: 10.17179/excli2022-4976 (PMC9360474; doi:10.17179/excli2022-4976)
Supplement: Supplementary information [file EXCLI-21-818-s-001.pdf]

EXCLI Journal 2022;21:818-839 – ISSN 1611-2156
Received: April 21, 2022, accepted: June 14, 2022, published: June 20, 2022

Supplementary information to:

Review article:

THE ASSOCIATION OF *ACE1*, *ACE2*, *TMPRSS2*, *IFITM3* AND *VDR* POLYMORPHISMS WITH COVID-19 SEVERITY: A SYSTEMATIC REVIEW AND META-ANALYSIS

Zorana Dobrijević, Dragana Robajac, Nikola Gligorijević, Miloš Šunderić, Ana Penezić, Goran Miljuš, Olgica Nedić

University of Belgrade – Institute for the Application of Nuclear Energy (INEP), Belgrade, Serbia

* **Corresponding author:** Zorana Dobrijević, Department for Metabolism, Institute for the Application of Nuclear Energy (INEP), University of Belgrade, Belgrade, Serbia
Tel: +381 11 2618 666, E-mail: zorana.dobrijevic@inep.co.rs

https://dx.doi.org/10.17179/excli2022-4976

This is an Open Access article distributed under the terms of the Creative Commons Attribution License (http://creativecommons.org/licenses/by/4.0/).

EXCLI Journal 2022;21:818-839 – ISSN 1611-2156
Received: April 21, 2022, accepted: June 14, 2022, published: June 20, 2022

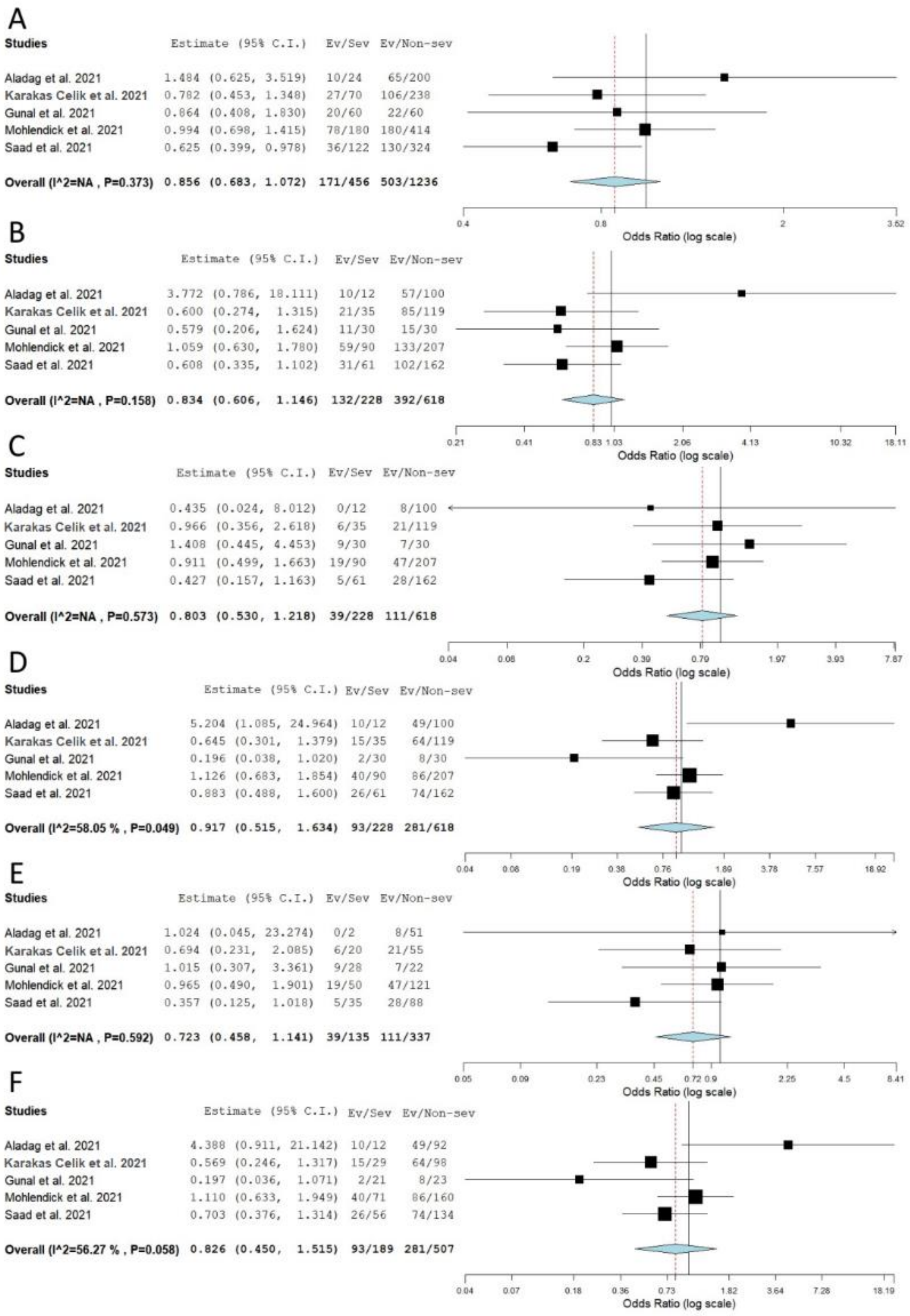

Supplementary Figure 1: Meta-analysis of the association between rs1799752 in *ACE1* and COVID-19 severity: comparison severe vs. non-severe. **A**) allelic model; **B**) dominant model; **C**) recessive model; **D**) overdominant model; **E**) II vs. DD; **F**) DI vs. DD. The results of the included studies presented as ORs, with 95 CI, and the overall effect with 95 % CI are shown in the forest plot. *P* values given are derived from heterogeneity tests.

EXCLI Journal 2022;21:818-839 – ISSN 1611-2156
Received: April 21, 2022, accepted: June 14, 2022, published: June 20, 2022

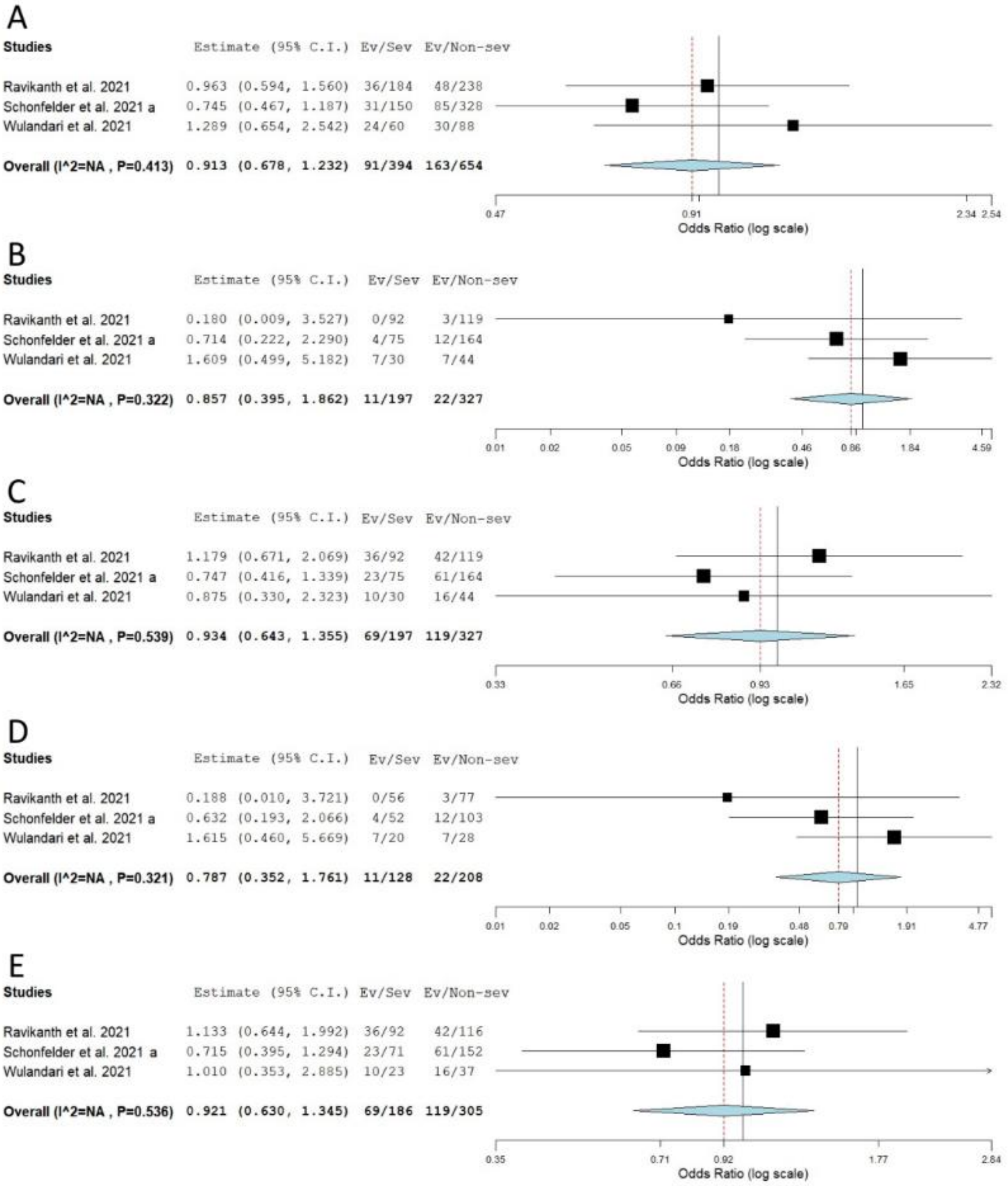

Supplementary Figure 2: Meta-analysis of the association between rs12329760 in *TMPRSS2* and COVID-19 severity, after the exclusion of asymptomatic SARS-CoV-2 infected participants: comparison severe vs. non-severe. **A**) allelic model; **B**) recessive model; **C**) overdominant model; **D**) AA vs. GG; **E**) GA vs. GG. The results of the included studies presented as ORs, with 95 % CI, and the overall effect with 95 % CI are shown in the forest plot. *P* values given are derived from heterogeneity tests.

EXCLI Journal 2022;21:818-839 – ISSN 1611-2156
Received: April 21, 2022, accepted: June 14, 2022, published: June 20, 2022

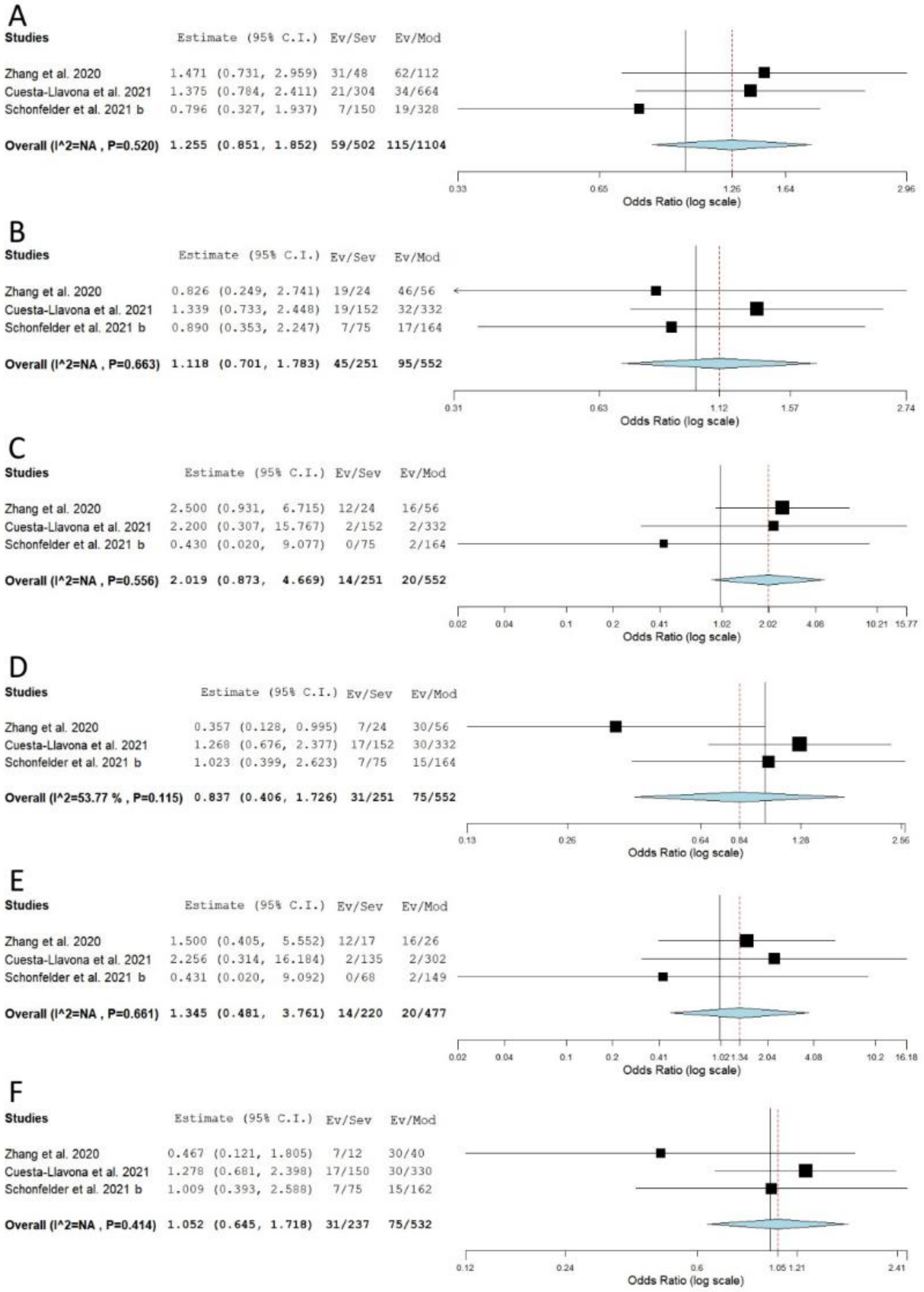

Supplementary Figure 3: Meta-analysis of the association between rs12252 in *IFITM3* and COVID-19 severity: comparison severe vs. moderate. **A**) allelic model; **B**) dominant model; **C**) recessive model; **D**) overdominant model; **E**) CC vs. TT; **F**) TC vs. TT. The results of the included studies presented as ORs, with 95 % CI, and the overall effect with 95 % CI are shown in the forest plot. *P* values given are derived from heterogeneity tests.

EXCLI Journal 2022;21:818-839 – ISSN 1611-2156
Received: April 21, 2022, accepted: June 14, 2022, published: June 20, 2022

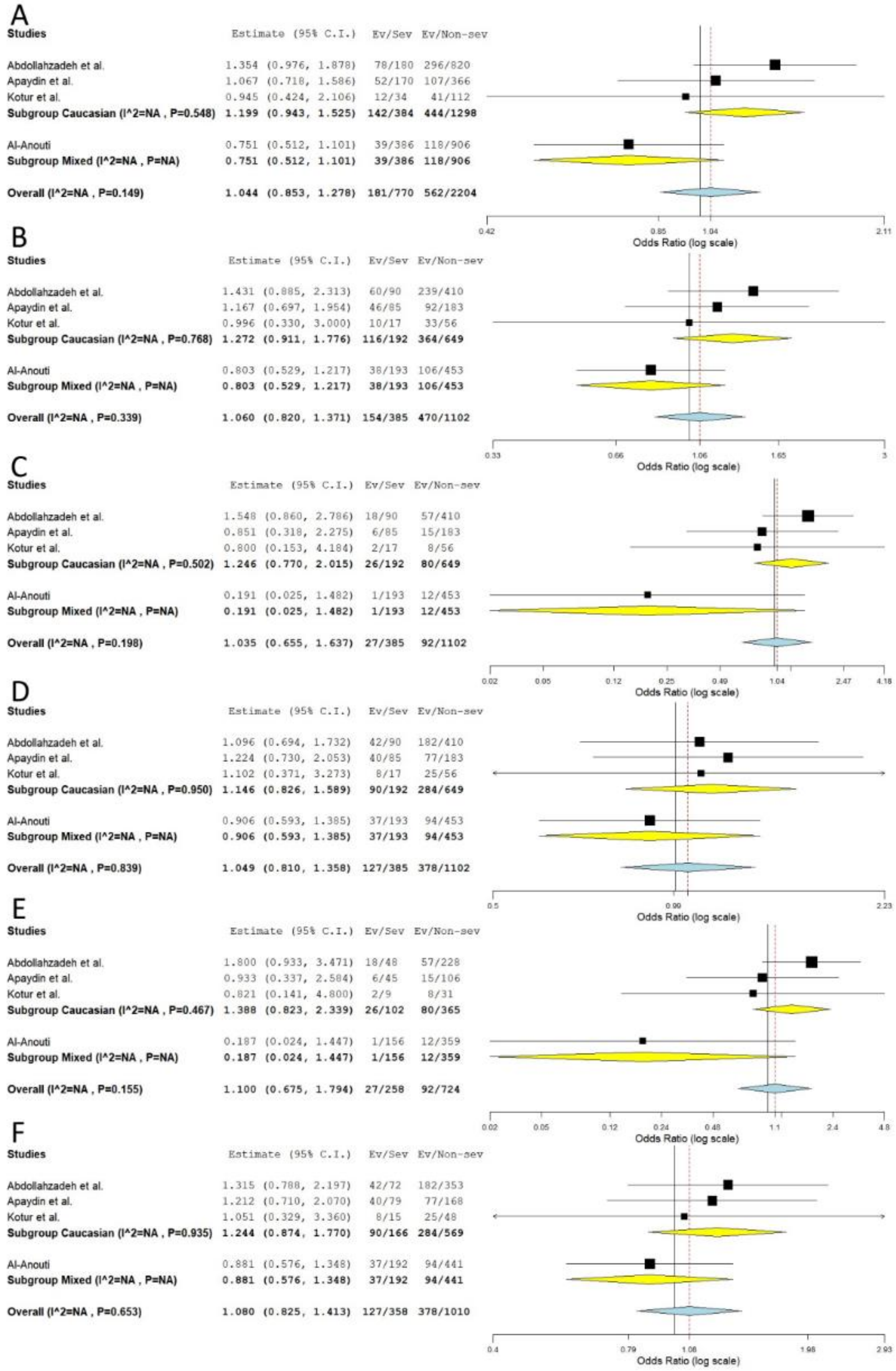

Supplementary Figure 4: Meta-analysis of the association between rs2228570 in *VDR* and COVID-19 severity: comparison severe vs. non-severe. **A**) allelic model; **B**) dominant model; **C**) recessive model; **D**) overdominant model; **E**) AA vs. GG; **F**) GA vs. GG. The results of the included studies presented as ORs, with 95 % CI, and the overall effect with 95 % CI are shown in the forest plot. *P* values given are derived from heterogeneity tests.

EXCLI Journal 2022;21:818-839 – ISSN 1611-2156
Received: April 21, 2022, accepted: June 14, 2022, published: June 20, 2022

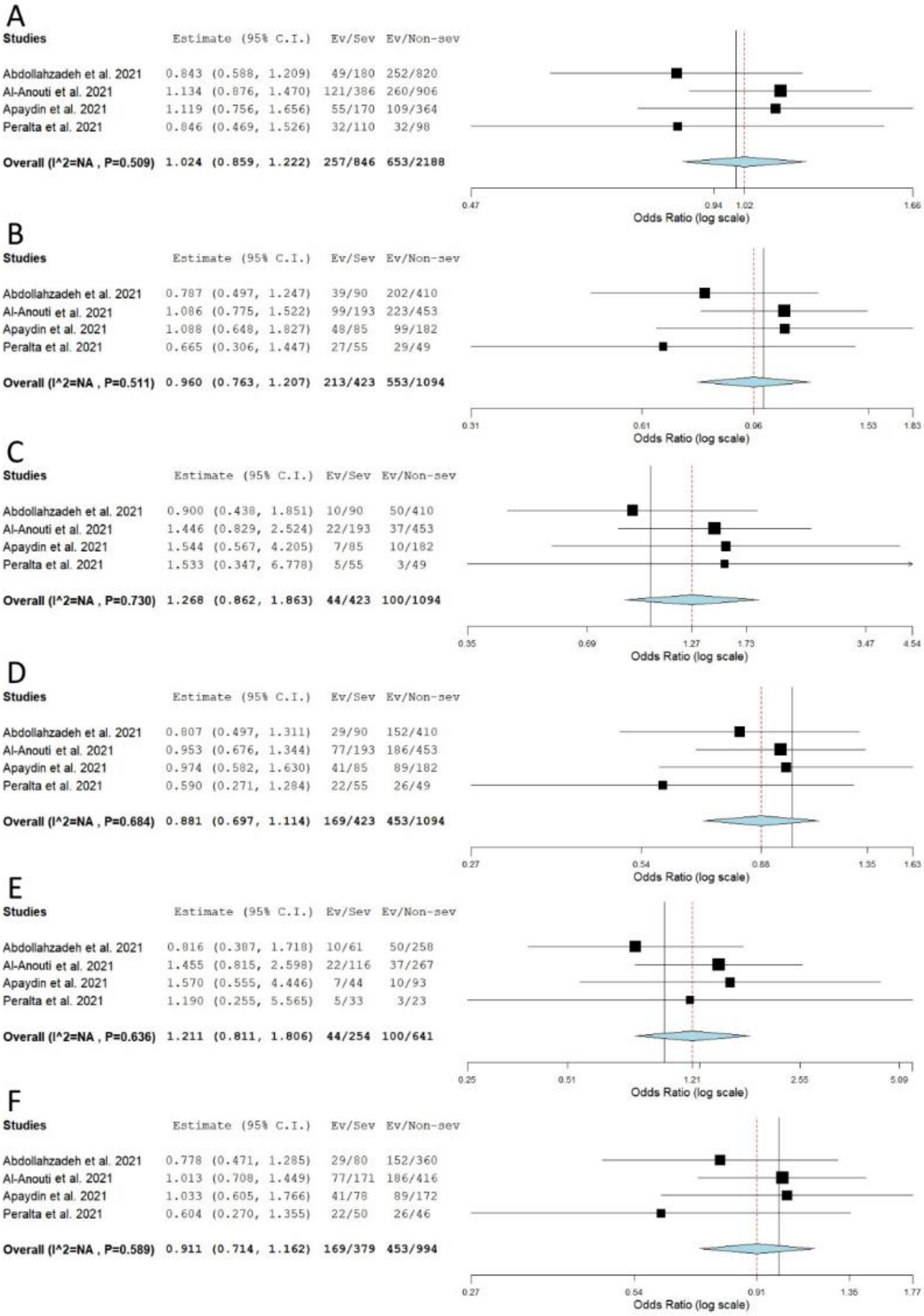

Supplementary Figure 5: Meta-analysis of the association between rs731236 in *VDR* and COVID-19 severity: comparison severe vs. non-severe. **A**) allelic model; **B**) dominant model; **C**) recessive model; **D**) overdominant model; **E**) GG vs. AA; **F**) AG vs. AA. The results of the included studies presented as ORs, with 95 % CI, and the overall effect with 95 % CI are shown in the forest plot. *P* values given are derived from heterogeneity tests.

EXCLI Journal 2022;21:818-839 – ISSN 1611-2156
Received: April 21, 2022, accepted: June 14, 2022, published: June 20, 2022

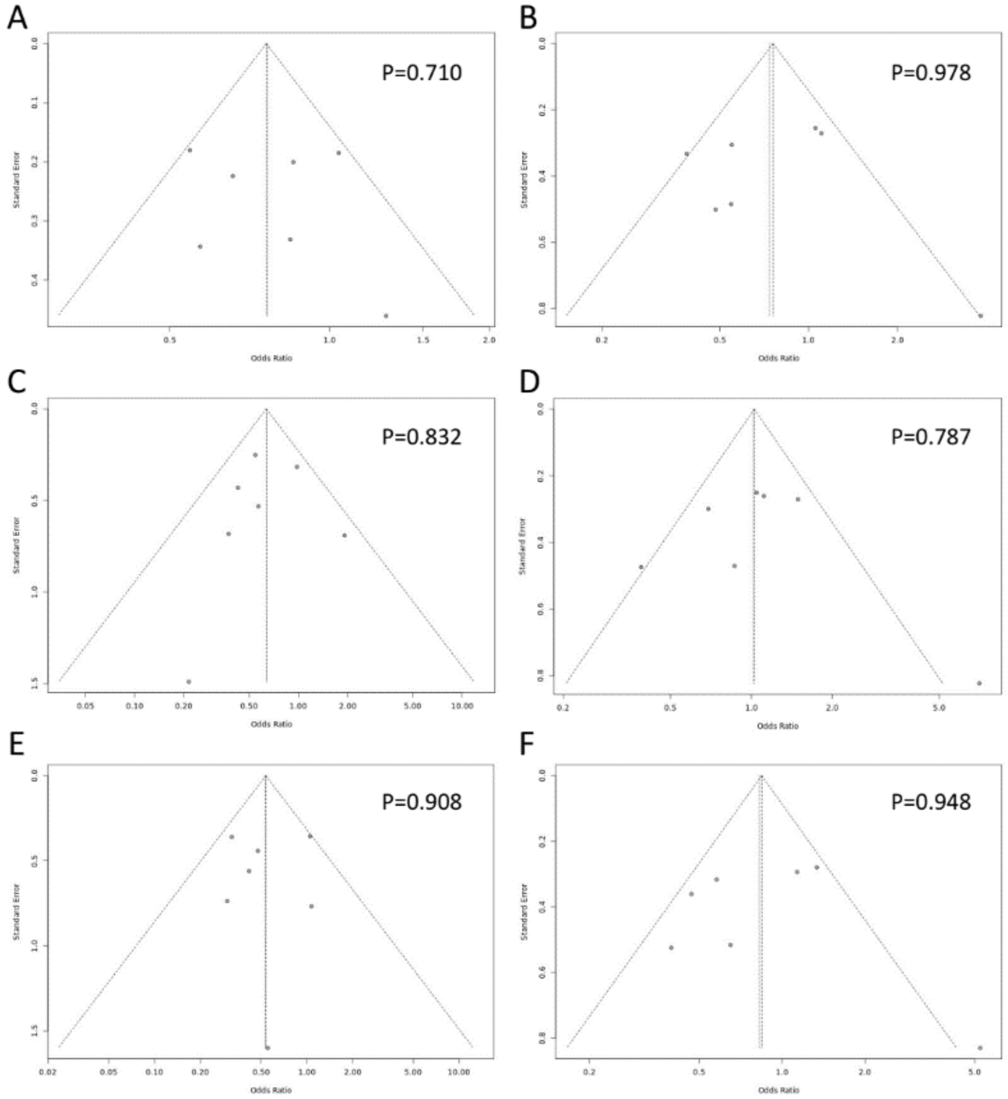

Supplementary Figure 6: Funnel plots for meta-analyses of the association between rs1799752 in *ACE1* and COVID-19 severity: comparison severe vs. moderate. **A**) allelic model; **B**) dominant model; **C**) recessive model; **D**) overdominant model; **E**) II vs. DD; **F**) DI vs. DD. *P* values presented are derived from Egger's tests.

EXCLI Journal 2022;21:818-839 – ISSN 1611-2156
Received: April 21, 2022, accepted: June 14, 2022, published: June 20, 2022

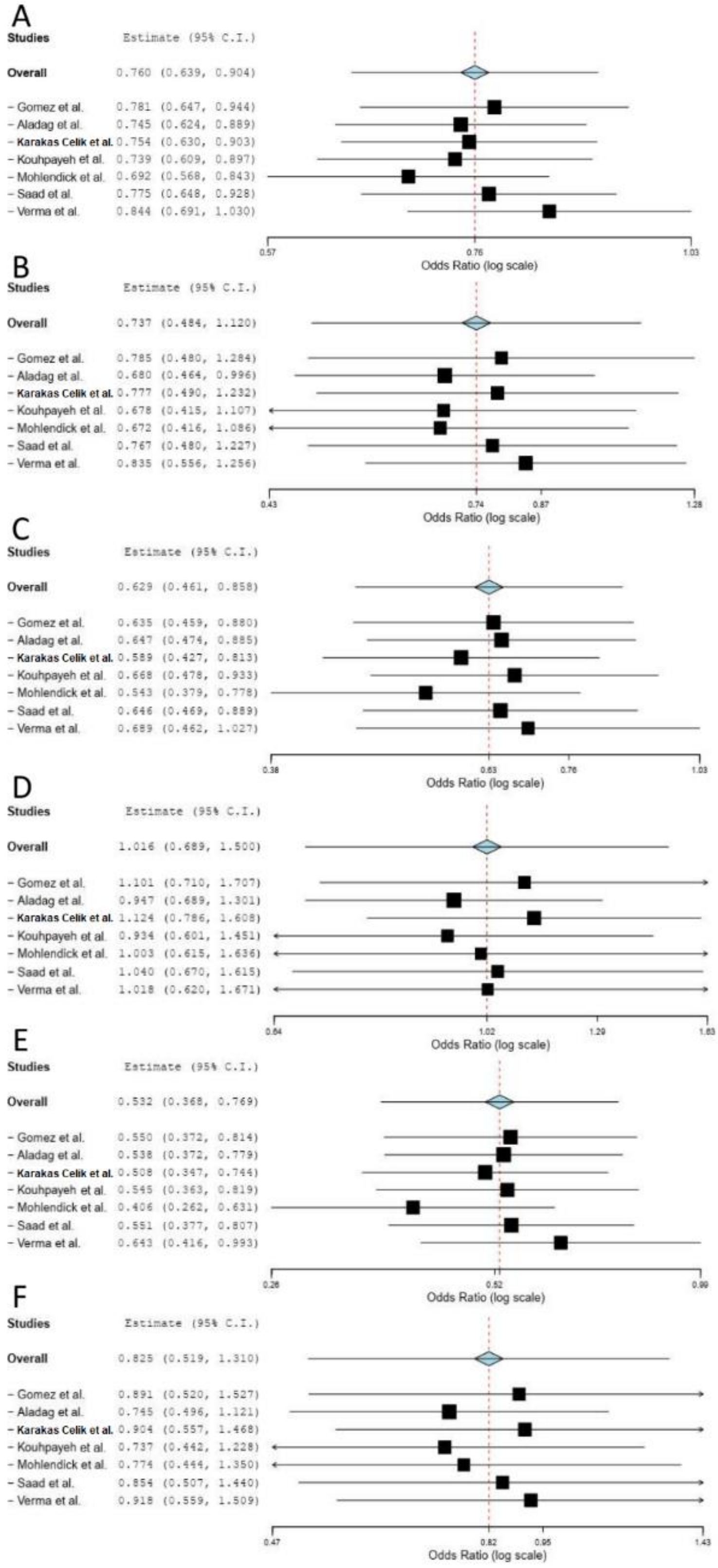

Supplementary Figure 7: Forest plots representing the results of sensitivity testing by leave-one-out meta-analysis of the association between rs1799752 in *ACE1* and COVID-19 severity: comparison severe vs. moderate. **A**) allelic model; **B**) dominant model; **C**) recessive model; **D**) overdominant model; **E**) II vs. DD; **F**) DI vs. DD. ORs with their 95 % CIs were used as risk estimates.

EXCLI Journal 2022;21:818-839 – ISSN 1611-2156
Received: April 21, 2022, accepted: June 14, 2022, published: June 20, 2022

Supplementary Table 1: Newcastle-Ottawa scale quality assessment of the studies included in the qualitative synthesis

Authors*	Year	Selection				Comparability	Outcome			Score
		Item 1	Item 2	Item 3	Item 4	Item 5	Item 6	Item 7	Item 8	
**Gómez et al.**	2020	*	*	*	*	*	*	*	*	8
**Zhang et al.**	2020	*	*	*	*	*	*	*	*	8
**Abdollahzadeh et al.**	2021	*	*	*			*	*	*	6
**Akin et al.**	2022	*	*	*	*	*	*	*	*	8
**Aladag et al.**	2021	*	*	*	*	*	*	*	*	8
**Al-Anouti et al.**	2021	*	*	*	*	**	*	*	*	9
**Alghamdi et al.**	2021	*	*	*	*	**	*	*	*	9
**Apaydin et al.**	2021	*	*	*	*	*	*	*	*	8
**Cafiero et al.**	2021	*	*	*	*	*	*	*	*	8
**Cuesta-Llavona et al.**	2021	*	*	*	*		*	*	*	7
**Gómez et al.**	2021	*	*	*	*	**	*	*	*	9
**Gunal et al.**	2021	*	*	*	*		*	*	*	7
**Hubacek et al.**	2021	*	*	*	*		*	*	*	7
**Íñiguez et al.**	2021	*	*	*	*	**	*	*	*	9
**Karakaş Çelik et al.**	2021	*	*	*	*		*	*	*	7
**Kotur et al. (adults)**	2021	*	*	*	*	**	*	*	*	9
**Kouhpayeh et al.**	2021	*	*	*	*	**	*	*	*	9
**Mir et al.**	2021	*	*	*	*		*	*	*	7
**Möhlendick et al.**	2021	*	*	*	*		*	*	*	7
**Monticelli et al.**	2021	*	*	*			*	*	*	6
**Peralta et al.**	2021	*	*	*	*		*	*	*	7
**Ravikanth et al.**	2021	*	*	*	*	**	*	*	*	9
**Saad et al.**	2021	*	*	*	*	**	*	*	*	9
**Schönfelder et al.**	2021a	*	*	*	*		*	*	*	7
**Schönfelder et al.**	2021b	*	*	*	*		*	*	*	7
**Verma et al.**	2021	*	*	*	*	**	*	*	*	9
**Wulandari et al.**	2021	*	*	*	*		*	*	*	7
**Akbari et al.**	2022	*	*	*	*	**	*	*	*	9
**Akin et al.**	2022	*	*	*	*	*	*	*	*	8
**Wang et al.**	2022	*	*	*	*		*	*	*	7

* References see main document
